# MicroRNA-1 and Circulating Microvesicles Mediate the Protective Effects of Dantonic in Acute Myocardial Infarction Rat Models

**DOI:** 10.3389/fphys.2018.00664

**Published:** 2018-09-24

**Authors:** Tingting Sun, Lihua Zhang, Xinxin Li, Fengfei Chen, Yanchuan Li, Xiaohui Ma, Feng Yu

**Affiliations:** ^1^School of Basic Medicine and Clinical Pharmacy, China Pharmaceutical University, Nanjing, China; ^2^State Key Laboratory of Core Technology in Innovative Chinese Medicine, Pharmacology and Toxicology Research Centre, Tasly Academy, Tasly Holding Group Co., Ltd., Tianjin, China

**Keywords:** dantonic, microRNA-1, microvesicles, acute myocardial infarction, protective effects

## Abstract

**Aim:** To investigate the protective effect of dantonic in ischemic myocardial damage by evaluating the expression of circulating microvesicles (MVs) and microRNA-1 (miR-1) in two animal models.

**Methods:** Two animal models of myocardial ischemia were established that were isoproterenol-induced myocardial ischemia (ISO-AMI) rat model and the acute myocardial infarction rat model induced by ligation of the left anterior descending coronary artery (LAD-AMI) of rat. To investigate the protective effect of dantonic, we observed the myocardial infarction size, creatine kinase (CK), lactate dehydrogenase (LDH), aspartate aminotransferase (AST) activities, cardiac troponin I (cTnI) level in serum, and the plasma levels of miR-1 and MVs.

**Results:** The results showed that pretreatment with dantonic significantly attenuated the LAD-AMI induced myocardial damage by decreasing the size of myocardial infarction, CK, LDH, AST activities, and cTnI level in serum. High dose dantonic treatment could significantly abrogate the increased plasma levels of miR-1 and MVs as compared to the LAD rat model. In addition, pretreatment with dantonic also showed a significant myocardial protective effect through reducing the expression levels of CK, LDH, and AST as compared to the ISO-AMI model. Whereas the cTnI level was no significant difference between model group and control group, suggesting that the model caused less myocardial damage. In the ISO-induced myocardial ischemia model, there is no significant difference between the model group with the control group of MVs and miR-1 levels. This may be that miR-1 is reported as a biomarker of acute myocardial infarction. The pathological changes of IOS-induced acute myocardial ischemia model are also different from those of acute myocardial infarction.

**Conclusion:** Dantonic showed the protective effect in these two ischemic myocardial injury rat models, whereas the circulating miR-1 and MVs levels were only ameliorated in the LAD rat model.

## Introduction

Acute myocardial infarction remains a leading cause of morbidity and mortality in the most developed countries worldwide. It has been estimated that an American suffers from an AMI experience approximately every 42 s (Mozaffarian et al., 2016). According to the Chinese Cardiovascular Disease Report 2016, the number of patients with cardiovascular disease in China is about 290 million, among which the 11 million are with coronary heart disease, acute myocardial infarction mortality rate of 110 people/100,000 people. Various chemical drugs, such as β-blockers, nitrate preparations, and calcium antagonists, have been used for treatment of AMI. However, long-term use of these chemical drugs might result in significant side effects, such as hypertension and bradycardia (Hlatky et al., 2014). Whereas the significant effects of traditional Chinese medicines (TCM) are becoming more popular (Xin et al., 2013), and they are particularly advantageous for treatment of AMI (Liu et al., 2011).

Dantonic, consisting of Salvia miltiorrhiza (SM), Panax notoginseng (PN), and Borneol, is a widely used TCM for treating ischemic angina pectoris, and has recently finished the phase III clinical trial assessment by the US Food and Drug Administration in 2016, and the results showed Dantonic safe and effective treatment of chronic stable angina. Compared with placebo control group and notoginseng borneol group had statistically significant (*P* < 0.05). The clinical value of Dantonic in the treatment of chronic stable angina pectoris was confirmed again by Phase III clinical trial of FDA. Previous studies showed that Dantonic treatment could significantly reduce the ischemia/reperfusion- (I/R-) and LAD induced myocardial damage and apoptosis, myocardial fibrosis, microcirculatory disturbance (Zhao et al., 2010; Wei et al., 2013; Yang et al., 2013), oxidative stress, and inflammatory response (Horie et al., 2005; Lee et al., 2013; Jun et al., 2014; Ren-an et al., 2014; Chen et al., 2016; Xu et al., 2016).

Many studies focused on the mechanism of AMI by which the circulating microRNAs (miRNAs) takes an active part in the regulation of myocardial infarction (Sala et al., 2014). In AMI, circulating cardiac extracellular vesicles (EVs) contain abundant cardiac-specific miRNAs that serve as indicators of cardiac damage. Among these miRNAs, microRNA-1 (miR-1) is one of the most abundant miRNAs in heart along with a heart- and muscle-specific miRNA. Several reports have indicated that miR-1 is upregulated in serum/plasma or urine by AMI and can be used as a diagnostic biomarker of AMI (Ai et al., 2010; Cheng et al., 2010; Kuwabara et al., 2011; Long et al., 2012). Microvesicles (MVs) as a carrier of circulating cell-free miRNAs, is one member of EVs family. Researchers discovered that MVs could be up-regulated by damaged cardiac muscle cells, which reflected an adaptive response to indicate other cells about heart injury. Hence, this present study is aimed to demonstrate the effect of Dantonic on plasma levels of miR-1 and MVs in AMI models.

## Materials and Methods

### Animals

Male Sprague-Dawley rats weighing 250 ± 20 g obtained from Beijing Vital River Laboratory Animal Technology Co., Ltd. [Beijing, China, Permission No. SCXK (Jing) 2012-0001] were used in this study. Rats were housed in a humidity of 40% ± 5% and a temperature of 22°C ± 2°C under a 12/12h light/dark cycle. Rats were free to access water and food, while fasted for 12 h before the AMI surgery. All animal experiments were approved by the Animal Care and Use Committee of Tasly (TSL-IACUC-2016-007).

### Agents

Dantonic was purchased from Tasly Pharmaceutical Co., Ltd. (Tianjin, China). An analysis by high performance liquid chromatography was carried out for quality control of Dantonic, with one pill containing 9 mg of SM, 1.76 mg of PN, 0.5 mg of Borneol, and 13.74 mg of polyethylene glycol. Isoproterenol was bought from Tokyo Chemical Industry (Tokyo, Japan). Triphenyl tetrazolium chloride (TTC) was bought from Sigma-Aldrich Chemical Co., Ltd. (United States). TTC was dissolved in phosphate buffer at a concentration of 1%. The Elisa determination kit for cardiac troponin I (cTnI) was supplied by the Nanjing Jiancheng Bioengineering Institute (Nanjing, China). MiRNeasy Kit and miRNeasy Spike-In Control were purchased from QIAGEN Co., Ltd. (Germany). RNA PCR Kit and predesigned gene-specific primers were purchased from GeneCopoeia Inc. (United States).

### Animal Model

Two rat models were established in this experiment including isoproterenol-induced myocardial ischemia (ISO-AMI) and the acute myocardial infarction rat model induced by ligation of the left anterior descending coronary artery (LAD-AMI) of rat. Isoproterenol was dissolved in physiological saline and injected subcutaneously to rats (85 mg/kg) daily for two consecutive days to induce experimental myocardial ischemia (Wang et al., 2009). The control group was given with physiological saline. Rats of LAD-AMI model were anesthetized by an intraperitoneal injection of 20% urethane prior to surgery. A 6–0 silk suture was placed between the pulmonary and left auricle where the LAD is located. The successful induction of AMI was confirmed based on the observation of the pale color of the anterior portion of the left ventricle. In the sham group, a silk suture was passed through the myocardium without occluding the LAD.

### Experimental Protocols

Rats were randomly divided into four groups in each model including control/sham group, model group (ISO-AMI or LAD-AMI), dantonic low dose group (dantonic low + ISO or dantonic low + LAD), dantonic high dose group (dantonic high + ISO and dantonic high + LAD). The animals in the treatment groups were pretreated with dantonic by receiving the drug daily by gavage for 5 days. On days 4 and 5, isoproterenol was injected subcutaneously. Dantonic at the dose of 167.4 mg/kg/day (low dose group) and 502.2 mg/kg/day (high dose group) were respectively onefold and threefold more than the corresponding equivalently effective doses used in clinic. One and a half hour after the last administration of dantonic or water on day 5, animals were anesthetized and subjected to surgical procedure. In control/sham and model group, rats received the same amount of distilled water in the same way.

### TTC Staining

Blood samples were obtained from abdominal aorta after occluding LAD for 4 h on day 5. Hearts were removed and sliced transversely into five sections (1 mm thick) starting from the apex. The slices were incubated with 1% TTC at 37°C for 10 min under dark conditions to delineate the infarction area. The infarct heart area was represented by unstained (white) color whereas normal heart tissues were stained with red color. Infarction status was expressed as a weigh ratio: infarct area (white)/whole heart.

### Biochemical Studies

Cytosolic enzyme creatine kinase (CK), lactate dehydrogenase (LDH), and aspartate aminotransferase (AST) in serum were measured with autobiochemical analyzer (Hitachi, Ltd., Tokyo, Japan). Serum cTnI activity was detected by enzyme-linked immunoabsorbent assay (ELISA) methods using commercial kits.

### MVs Isolation and Flow Cytometric Analysis

Blood samples were centrifuged at 2,600 *g* for 15 min at room temperature to obtain platelet-poor plasma (PPP), which was then centrifuged at 10,000 *g* for 5 min at room temperature to obtain platelet-free plasma (PFP). PFP was subjected to the ultracentrifugation at 33,000 rpm for 150 min at 4°C, to harvest the pelleted MVs, which were then resuspended in 100 μl 0.9 % sodium chloride and kept at -20°C (Liu et al., 2015).

Dot plots of forward scatter (FSC) vs. side scatter (SSC) showed that 1, 2 μm standard beads were distributed in the appropriate region by modulating the photomultiplier tube (PMT) voltage. R1 area was gated under the region of 1 μm beads which was defined as MVs population. MVs in the R1 gate were further identified by fluorescent (FITC) labeled annexin V.

### Quantitative Real-Time RT-PCR Analysis

Total RNA samples from plasma were prepared using miRNeasy kit according to the manufacturer’s instruction. Samples of total small RNAs were reverse-transcribed using the miRNA qRT-PCR Detection Kit, and the resulting cDNA was used as a PCR template. The miRNA levels were determined by real-time PCR with the Stratagene Mx3000P Sequence Detector (Agilent, United States) according to the manufacturer’s instruction. miR-1 primer was designed and verified by GeneCopoeia Inc. The miRNeasy Spike-In Control is a *Caenorhabditis elegans* miR-39 miRNA was amplified as an internal control. All chemical reagents were used in this experiment were analytical grade. The relative miR-1 expression level was calculated using the comparative ΔCt method formula: 2^-ΔΔCt^.

### Statistical Analysis

Data were expressed as mean ± SEM. GraphPad Prism 5.0 program was applied for One-way ANOVA analysis for the statistical comparisons among the groups by using Tukey’s test. *P*-values less than 0.05 were considered as statistically significant.

## Results

### Dantonic Administration Exerting Beneficial Effects in the LAD-AMI Rat Model

**FIGURE 1:**
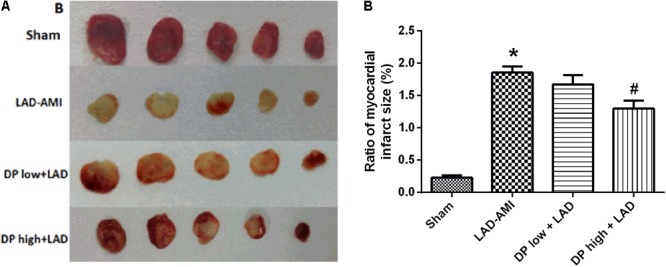
Effects of dantonic on rat myocardial infarct size. **(A)** Representative slices of ventricle stained by TTC in LAD-AMI model. **(B)** Quantitative analysis of infarct size-weight ratio: infarct area (white)/whole heart. Sham: control group;0020LAD-AMI: ligate LAD group; dantonic low + LAD: ligate LAD plus pre-treatment with dantonic 167.4 mg/kg/day group; dantonic high + LAD: ligate LAD plus pre-treatment with dantonic 502.2 mg/kg/day group. Data are presented as mean ± SEM. *n* = 10/group ^∗^*p* < 0.05 vs. Sham group, ^#^*p* < 0.05 vs. Model group.

The infarct weight ratio of each sample was detected to evaluate the cardio protective role of dantonic. Representative heart slices stained by TTC to delineate infarct size are shown in **Figure 1A**. Apparently, no infarct was observed in myocardial tissue slices from sham group. However, myocardium sections from model group exhibited obvious infarct areas, and administration of dantonic at low and high doses diminishes AMI-induced infarct ratio. Quantitative analysis of the infarct further confirmed that the hearts from dantonic-treated rats showed a significantly smaller infarct weight ratio than those from the model group (*P* < 0.05, **Figure 1B**), suggesting that dantonic administration exerted beneficial effects in LAD-AMI model.

### Dantonic Administration Decreasing Serum CK, LDH, and AST Activities

**FIGURE 2:**
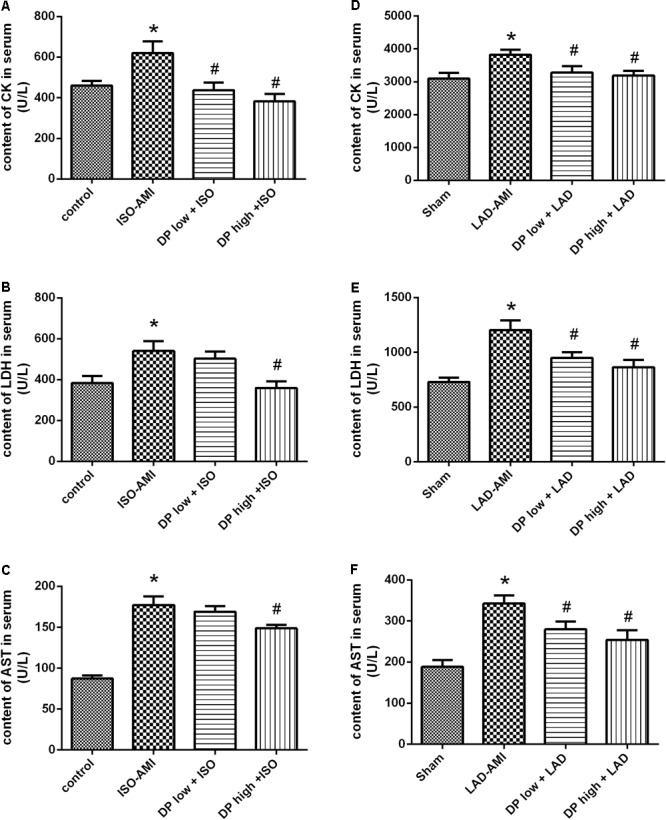
Effects of dantonic on rat serum marker enzymes activities. **(A,D)** Effect of dantonic on serum creatine kinase (CK) activity against isoproterenol-induced myocardial ischemia (ISO-AMI) and left anterior descending coronary artery (LAD-AMI). **(B,E)** Effect of dantonic on serum LDH activity against ISO-AMI and LAD-AMI. **(C,F)** Effect of dantonic on serum AST activity against ISO-AMI and LAD-AMI. Data are presented as mean ± SEM. ^∗^*p* < 0.05 vs. sham/control group, ^#^*p* < 0.05 vs. model group.

The activities of the serum marker enzymes CK, LDH, and AST were significantly increased in the ISO-AMI and LAD-AMI rat models compared to control and sham groups. While in the rats pre-treated with dantonic followed by isoproterenol administration or LAD ligation, the activities of the marker enzymes CK, LDH, and AST in the serum were decreased significantly. *n* = 10/group, *P* < 0.05, **Figures 2A–F**.

### Dantonic Administration Decreasing Serum cTnI Level in LAD-AMI

**FIGURE 3:**
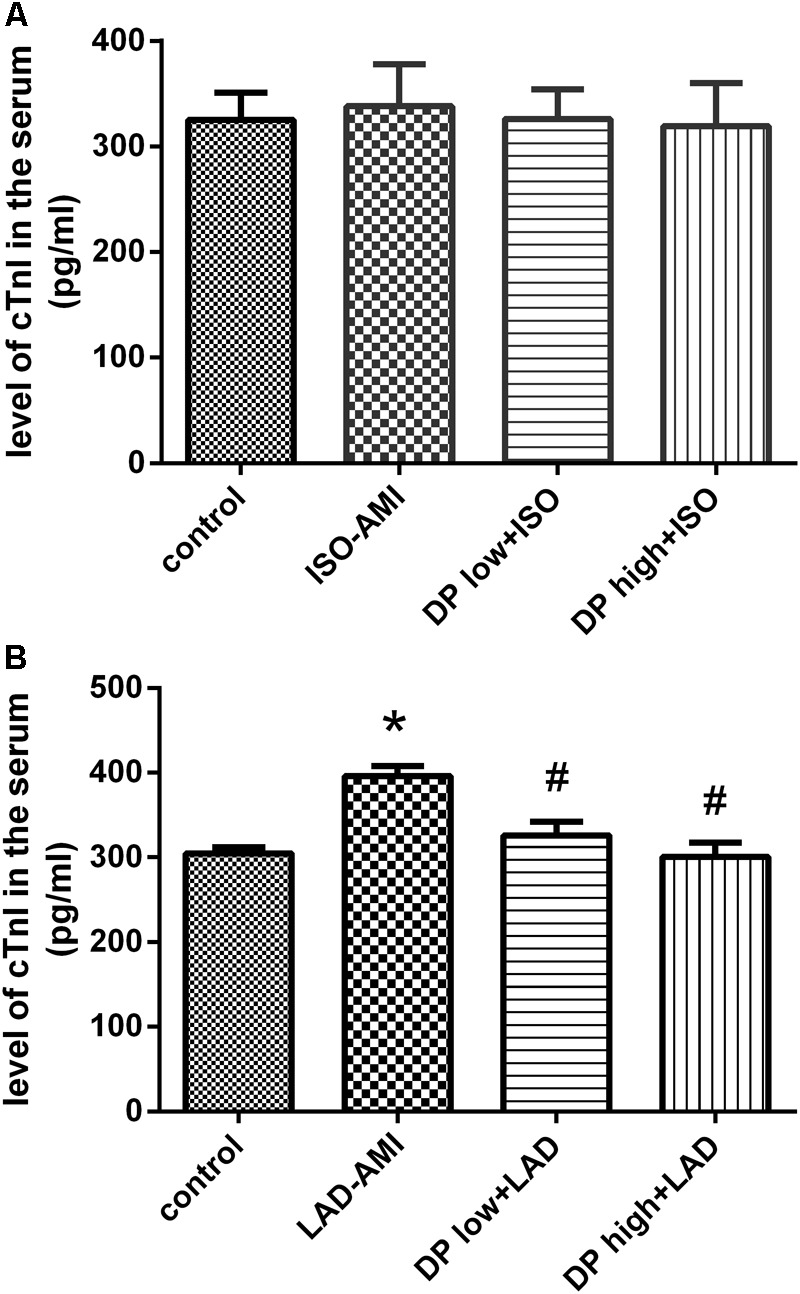
Effects of dantonic on rat serum cTnI activity. **(A)** Effect of Dantonic on serum cTnI activity against ISO-AMI. **(B)** Effect of Dantonic on serum cTnI activity against LAD-AMI. Data are represented as mean ± SEM. ^∗^*p* < 0.05 vs. Sham/control group, ^#^*p* < 0.05 vs. Model group.

The result shows that the level of cTnI in the serum. Rats induced with LAD showed significant elevation in the levels of cTnI in serum compared to control and sham rats. Pretreatment with dantonic showed a significant decrease in the level of serum cTnI when compared to the LAD-AMI groups. In ISO-AMI the level of serum cTnI has no significant between the control group and model group. (*n* = 10/group, *P* < 0.05, **Figure 3**).

### Dantonic Administration Decreasing MVs in the PFP Against LAD-AMI

**FIGURE 4:**
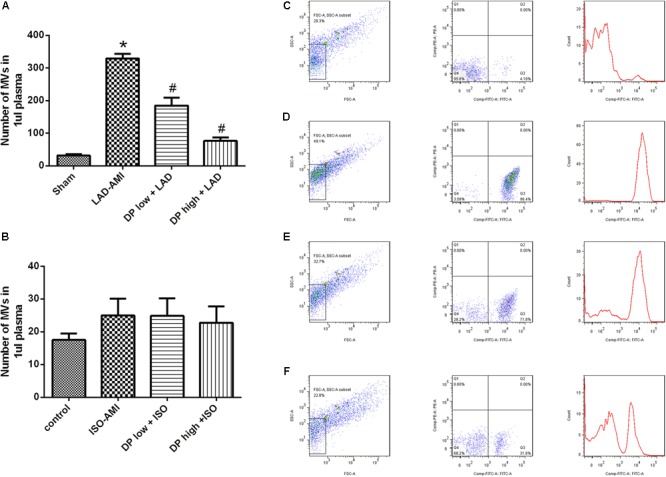
Effects of dantonic on the levels of rat plasma microvesicles (MVs). **(A)** Effect of dantonic on number of MVs in plasma (events/μl) against LAD-AMI. **(B)** Effect of dantonic on number of MVs in plasma (events/μl) against ISO-AMI. **(C)** Representative dot plots and histogram plot in sham group. Representative dot plots of forward scatter (FSC) vs. side scatter (SSC) showed that 1, 2 μm latex beads. MVs were identified as events with size less than 1 μm within the gate R1. **(D)** Representative dot plots and histogram plot in LAD-AMI group. **(E)** Representative dot plots and histogram plot in dantonic low + LAD group. **(F)** Representative dot plots and histogram plot in dantonic high + LAD group. Data are presented as mean ± SEM. *n* = 3/group ^∗^*p* < 0.05 vs. sham/control group, ^#^*p* < 0.05 vs. model group.

Microvesicles were significantly increased in the LAD rat model as compared to the sham group. Dantonic at high dose significantly reduced the plasma levels of MVs as compared to the model group (*P* < 0.05, **Figure 4A**); But there was no significant difference in the plasma level of MVs between ISO-AMI group and control group (**Figure 4B**). Representative flow cytometry dot plots and histogram plots in LAD model are shown in **Figures 4C–F**. (*n* = 6/group).

### Dantonic Administration Decreasing the Plasma Level of miRNA-1 Induced by LAD-AMI

**FIGURE 5:**
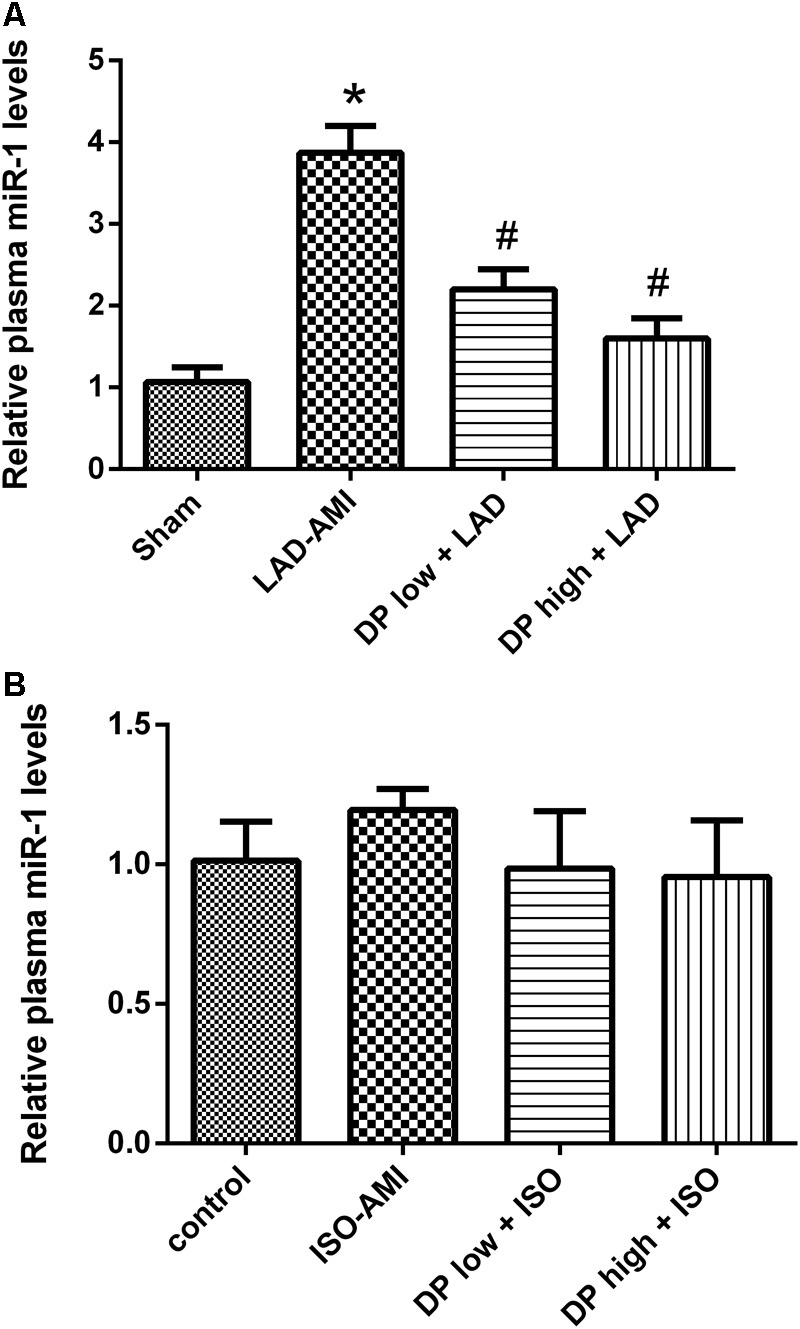
Effect of dantonic on plasma miRNA-1 level. **(A)** Effect of dantonic on plasma miRNA-1 level induced by LAD-AMI. **(B)** Effect of Dantonic on the plasma miRNA-1 level induced by ISO-AMI. Data are presented as mean ± SEM. ^∗^*p* < 0.05 vs. sham/control group, ^#^*p* < 0.05 vs. model group.

The quantitative Real Time-PCR data demonstrated that ligate LAD could obviously increase the plasma level of miR-1 as compared to sham group, and the miR-1 level was significantly lower in both dantonic-administrated groups compared to LAD-AMI group (*P* < 0.05, **Figure 5A**). But there was no significant difference in plasma level of miRNA-1 between ISO-AMI group and control group (*n* = 3/group) (**Figure 5B**).

## Discussion

Dantonic has been widely applied for prevention and treatment of angina, myocardial ischemia and other cardiovascular diseases in the clinical. More than 450 million people have taken dantonic since it was on the market in 1994. Besides, dantonic has been marketed in many countries (e.g., Canada, Singapore, The United Arab Emirates, Korea, Russia, Cuba, Vietnam, India, and South Africa) as a dietary supplementary for the prevention and treatment of ischemic heart diseases (Chu et al., 2011). Previous studies revealed that the pharmacological mechanism of dantonic against AMI involved anti-platelet aggregation, reducing the overload of calcium and circulating adhesion molecules, ameliorating myocardial fibrosis, protecting against microcirculatory disturbances, inhibiting NADPH oxidase, and modulating the perturbed energy metabolism (Xin et al., 2013; Yang et al., 2013; Zou et al., 2015).

It was discovered that the endogenous plasma miRNAs could be protected in some manner through packaging in microparticles (exosomes, MVs, and apoptotic bodies) or associating with RNA-binding proteins (Argonaute2) or lipoprotein complexes (high-density lipoprotein) to prevent their degradation (Creemers et al., 2012). MVs contain more than 1200 miRNAs and approximately 121 miRNAs can be delivered from one cell to another. Circulating miRNA may be physiologically active and play a pivotal role in cell-to-cell communication as well as MVs. Among these miRNAs, miR-1, miR-133a, miR-208, and miR-499 are muscle-enriched which are crucial to regulate the metabolism of the myocardium. Several reports have indicated that miR-1 is upregulated in serum/plasma or urine by AMI and can be used as a biomarker of AMI (Ai et al., 2010; Cheng et al., 2010; Long et al., 2012).

Most of the previous researches about muscle-enriched miRNAs were conducted on patients in clinical trials. But unlike the current study, this time we focused on the changes of miR-1 and MVs in the plasma of rats in the AMI model and dantonic treatment groups. Reports indicated that the circulating miRNAs derived from heart might originate from dead cells after MI and the miRNAs level could reflect the degree of myocardial injury. Additionally miR-1 levels were significantly correlated with the cTnI level in serum (Kuwabara et al., 2011). These are consistent with our experimental results conducted on the LAD-AMI model. The results of our study revealed that pretreatment with dantonic significantly attenuated the AMI-induced myocardial damage by decreasing the myocardial infarction size, CK, LDH, AST activities, and the cTnI level in serum. Dantonic at high dose could significantly abrogate the increase in the plasma levels of miR-1 and MVs induced by LAD-AMI.

Subcutaneous injection of isoproterenol can establish the myocardial ischemia animal model. ISO in supramaximal doses induces morphological and functional alterations in heart and then generates highly cytotoxic free radicals through auto-oxidation of catecholamines, which has been implicated as one of the important causative factors in isoproterenol-induced myocardial damage. It has been reported that auto-oxidation of excess catecholamines results in free radical mediated peroxidation of membrane phospholipids and consequently leads to the permeability changes in the myocardial membrane, intracellular calcium overload and irreversible damage. But miR-1 targets e-NOS, PKC𝜀, HSP60, and regulates muscle cell differentiation, cardiac hypertrophy (Sahoo and Losordo, 2014). In this study, the results show that only CK and LDH levels were significantly increased in the ISO induced myocardial ischemia rat model as compared with the control group, whereas dantoic high dose group could significantly reduce the serum content (*P* < 0.05). Other indicators including cTnI, microRNA, microcapsules were not significantly different between the groups. The serum CK and LDH results were consistent with those reported in the literature. The cTnI results of the model group were lower than previous report. Microcapsules and miRNAs have not yet reported on this model, and the cause of this outcome may be due to the different mechanisms of myocardial injury with the AMI-LAD model.

In the present study, we confirmed that pretreatment with dantonic for 5 days significantly prevented myocardial damage induced by ISO-AMI and LAD-AMI. In addition, we reported for the first time the *in vivo* data demonstrated that pretreatment with dantonic significantly inhibited LAD-induced increase in miR-1 and MVs levels in plasma. MVs were shed by various cells, especially by platelets, endothelial cells and erythrocytes. MVs appeared to be produced in response to the stimuli (Cocucci et al., 2009) and were defined by their capacity to bind to annexin V, which is an adhesion molecule that specifically interacts with phosphatidylserine (Connor et al., 2010). At present, the form and mechanism of MVs are not very clear *in vivo* and the effect of dantonic on MVs from particular source needs further research.

## Author Contributions

TS is the first author and responses for the entire research. XL, LZ, FC, and YL are assistants of the research. XM and FY are corresponding authors of the article.

## Conflict of Interest Statement

LZ, XL, YL, and XM are employed by Tasly Holding Group Co., Ltd. TS and FC are dual cultured by China Pharmaceutical University and Tasly Holding Group Co., Ltd. The remaining author declares that the research was conducted in the absence of any commercial or financial relationships that could be construed as a potential conflict of interest.
